# A Nanoscale Shape-Discovery Framework Supporting Systematic Investigations of Shape-Dependent Biological Effects and Immunomodulation

**DOI:** 10.1021/acsnano.1c10074

**Published:** 2021-12-27

**Authors:** Wei Zhang, Hender Lopez, Luca Boselli, Paolo Bigini, André Perez-Potti, Zengchun Xie, Valentina Castagnola, Qi Cai, Camila P. Silveira, Joao M. de Araujo, Laura Talamini, Nicolò Panini, Giuseppe Ristagno, Martina B. Violatto, Stéphanie Devineau, Marco P. Monopoli, Mario Salmona, Valeria A. Giannone, Sandra Lara, Kenneth A. Dawson, Yan Yan

**Affiliations:** † Guangdong Provincial Education Department Key Laboratory of Nano-Immunoregulation Tumor Microenvironment, The Second Affiliated Hospital, Guangzhou Medical University, Guangzhou 510260, Guangdong P.R. China; ‡ Centre for BioNano Interactions, School of Chemistry, University College Dublin, Belfield, Dublin 4, Ireland; & School of Physics and Optometric & Clinical Sciences, Technological University Dublin, Grangegorman D07XT95, Ireland; ∥ Istituto di Ricerche Farmacologiche Mario Negri IRCCS, Via Mario Negri 2, 20156 Milan, Italy; ⊥ Departamento de Física Teórica e Experimental, Universidade Federal do Rio Grande do Norte, 59078970 Natal, RN, Brazil; # Department of Pathophysiology and Transplantation, University of Milan, 20122 Milan, Italy; g School of Biomolecular and Biomedical Science, UCD Conway Institute of Biomolecular and Biomedical Research, University College Dublin, Belfield, Dublin 4, Ireland

**Keywords:** nanoscale shape, shape identification, microfluidic, tunable synthesis, biological effects, immunomodulation

## Abstract

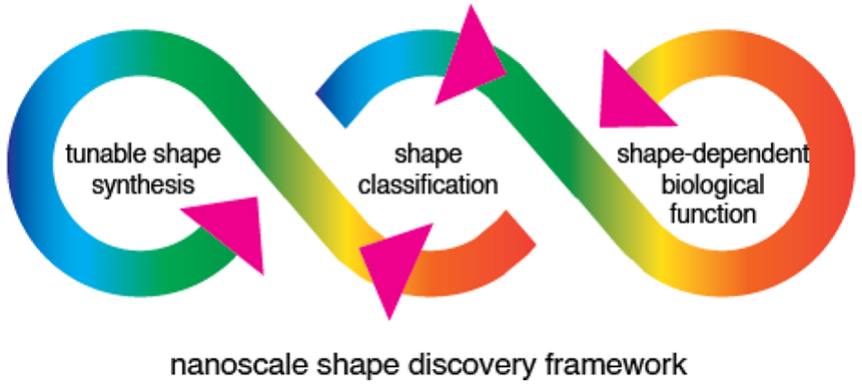

Since it is now possible to make, in a controlled fashion, an almost unlimited variety of nanostructure shapes, it is of increasing interest to understand the forms of biological control that nanoscale shape allows. However, *a priori* rational investigation of such a vast universe of shapes appears to present intractable fundamental and practical challenges. This has limited the useful systematic investigation of their biological interactions and the development of innovative nanoscale shape-dependent therapies. Here, we introduce a concept of biologically relevant inductive nanoscale shape discovery and evaluation that is ideally suited to, and will ultimately become, a vehicle for machine learning discovery. Combining the reproducibility and tunability of microfluidic flow nanochemistry syntheses, quantitative computational shape analysis, and iterative feedback from biological responses *in vitro* and *in vivo*, we show that these challenges can be mastered, allowing shape biology to be explored within accepted scientific and biomedical research paradigms. Early applications identify significant forms of shape-induced biological and adjuvant-like immunological control.

While the underlying principles and paradigms of nanostructure biological recognition and processing are quite different from those for biomolecules,^1−5^ this fundamental distinction is only beginning to be appreciated and applied in biology and medicine. This has resulted in much emphasis on the use of nanostructures as vehicles to “carry” drugs and other cargoes and more limited appreciation of the fundamental role that the nanostructure itself can play in biological control. Increasingly, we understand that, in contrast to molecular ligand–receptor binding, numerous interactions distributed across the whole nanostructure–cell interface (“synapse”) collectively induce a complex set of membrane and peri-membrane molecular events that we term “bionanoscale recognition”. In determining the nanostructure’s biological identity these processes take account of the details of the molecular presentation at the nanostructure’s surface,^3−6^ the detailed organization of nanoscale shape features and possibly other collective features yet to be discovered.^7−12^ Membrane signaling responses are sensitive to stress relaxational phenomena on the nanoscale, and since relaxation of peri-membrane recognition processes occurs on comparable length- and time scales, we hypothesize that correlated nanoscale shape features can be detected by the cell recognition machinery as patterns of differentiated stress relaxation at the membrane.^2^ However, extensive and detailed mechanistic investigations will be required to fully determine the mechanistic drivers of shape recognition on the nanoscale, greatly advanced and facilitated by recent advances in shape control and characterization.^6,8^

Clearly, for the field of shape-dependent biological effects to progress we now require concepts that will allow us to explore the science by systematic rather than (only) phenomenological trial and error investigations. Those of us seeking to develop this field of research see many competing priorities as to where to begin. In many ways we are like the early astronomers recording the apparently unlimited variety of events in the sky; somewhat awestruck by the infinitude of particle shapes and the extent of the nanoparticle shape universe, but unable as yet to make sense of the diversity and “meaning” (degree of biological control) that can be exerted by nanoscale shape biology. Certainly, the few early snapshots of shape biology we do have suggest an extraordinary richness of responses, distinct from simpler biological interactions, and hint at practical possibilities to control immunological, metabolic and other system level responses.^13−21^ Still for the field to grow we will need executable research programmes. First, it is important for shape characteristic data to be reportable and transferrable between different laboratories and across different approaches to the science. Investigators will need to use different types of reactors and syntheses but have transferrable structural inputs and biological outcomes in much the same way as we take for granted in small or biomolecular biological investigations. Clearly, the usual physiochemical data alone (while important) do not specify shape and are insufficient for this purpose. Shape quantification is required to make meaningful, reportable, and reproducible connections between shape distributions and biological outcomes. However, the major challenge is that in such a vast universe of shape we need to know where to look for interesting biological effects without the impracticalities of guessing or randomly searching a vast unknown shape space.

These and many other detailed technical features of nanoscale shape recognition have hitherto appeared to make systematic exploration of the relationship between nanoscale shape and biology a daunting proposition. For instance, (whether endogenous or man-made) even when the objective is to create a single shape identity, nanostructures are typically fabricated in weakly constrained assembly processes, leading to structural variations between individual particles and thereby to heterogeneous distributions. That, and the difficulty in controlling the process itself, often make it difficult to reproduce shape distributions and characterize them in a meaningful way. However, this challenge has now been addressed by advanced computational geometry methods applied to electron microscopy that digitize, capture, and analyze particle shape.^22−26^ Those methods now allow us to check the reproducibility of shape and dispersion characteristics of the ensembles and to develop methods to ensure those standards are met. Such advances now allow us to carry out meaningful, reportable and systematic biological investigations of nanoscale shape, if we know which shapes are of interest.

In this paper, we report on the next step, presenting a “shape discovery” approach that enables disciplined biological studies on interesting nanoscale shapes. Based on previous investigations of the mechanism behind the shape formation of branched GNPs, combining tunable microfluidic nanostructure flow synthesis capacities with a quantitative framework that captures and quantifies nanoscale shape, we are able to vary shape in a flexible manner, reproducibly making shape ensembles.^27^ Then coupling those microfluidic syntheses and digital shape characterization we use feedback from cellular (in the example discussed in this article: immune relevant) *in vitro* read-outs to inductively tune along a trajectory of different shape distributions to a regime of biological interest. Such inductively located particle ensembles are essentially “lead shape distributions” for further investigations. As a proof of concept, using this discovery process, we have identified an immunologically interesting nanoscale shape regime and confirmed the distinctive shape-dependent immunological properties by detailed analysis of antibody responses and B-cell receptor repertoire.

## Results and Discussion

### Definition and Characterization of Nanoscale Shape Ensemble Distributions

**Figure 1:**
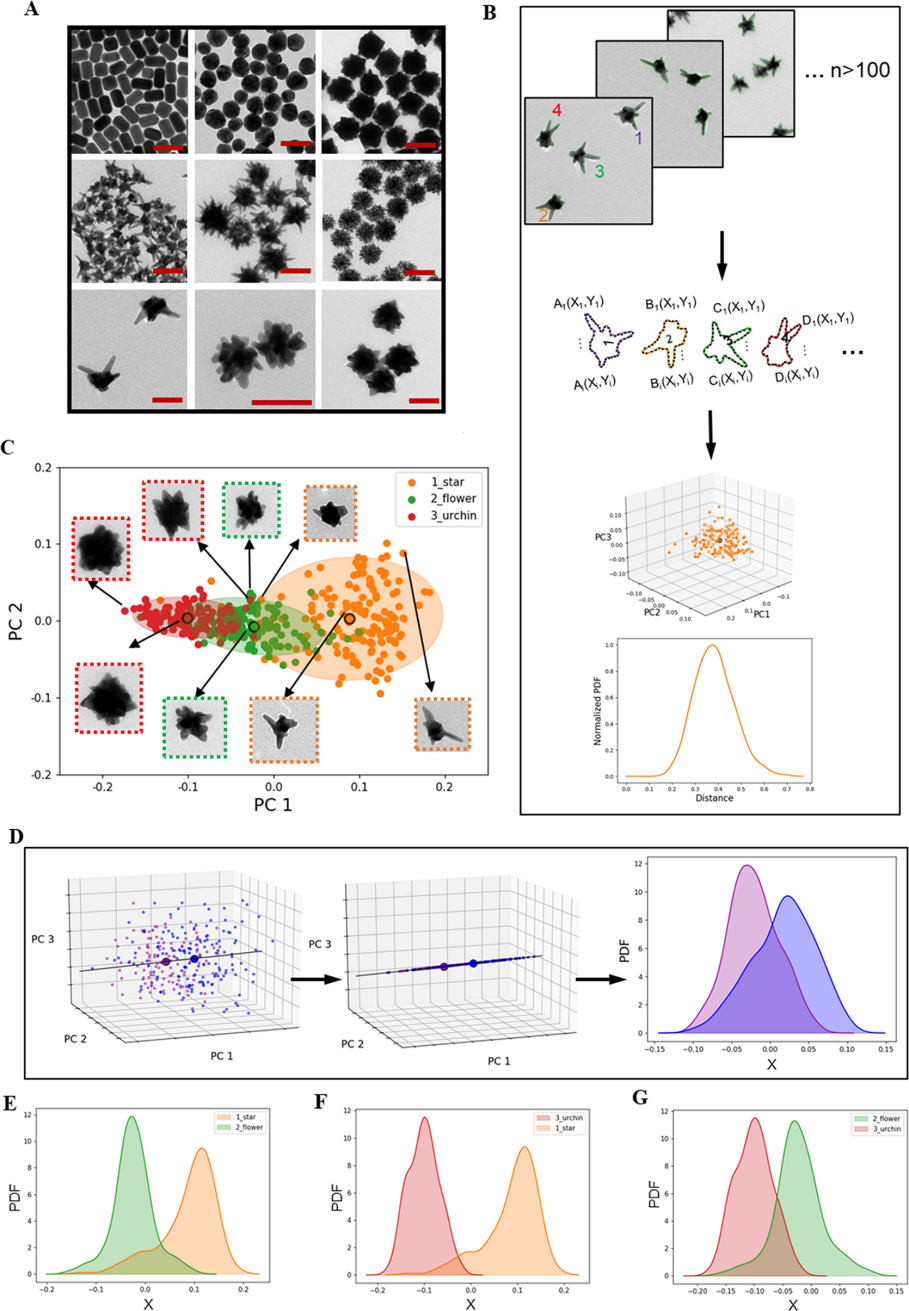
Definition of nanoscale shape ensemble distributions. (A) TEM micrographs showing the shape library of gold nanoparticles (GNPs), scale bar is 100 nm. (B) Schematic showing the process of nanoscale shape identification: capture and digitization of the contours of the nanostructures, shape space classification based on Fourier transform descriptor. (C) 2D scatter plot and TEM micrographs for selected points of the first two principal components (PC) obtained from the analysis of the shape descriptor for three nanostructures: star, flower, and urchin. The ellipses represent the regions which contain 95% of the points for each shape. (D) Schematic showing the process used to quantify the level of overlap between two nanoscale shapes. The coordinate X represents the line that joins the center of gravity of the two shapes in the 3D scatter plot of the first three principal components. The projected points onto the line X are used to calculate the probability distribution function (PDF) for each shape which is then used to measure the level of overlap between two shape distributions. Examples of the overlap quantification between star and flower (E), urchin and star (F), and flower and urchin (G).

While nanoscale shape ensembles have in the past been described using “typical” electron microscope images and evocative names (*e.g.*, stars, flowers, and urchins) (Figure 1A), here we use statistical nanostructure image capture, digitization, and quantitative computational analysis of shape ensembles. The key steps have been described before,^7^ including abstraction of minimal information capture of shape ensembles (*de facto* choosing a mathematical representation) and further condensation of that information (by principal component analysis) to allow it to be manipulated and analyzed, so here we only briefly summarize the features. Summarily, we first capture and digitize hundreds of nanostructure electron microscopy generated surfaces (or more condensed surface-projected contour descriptions) and, in much the same way signals are Fourier analyzed,^28−31^ transform the image contour of each particle into (typically) hundreds to thousands of discretized coefficients in a suitable representation (Figure 1B). At that point these coefficients are simply equivalent to the contour itself, but identification of the principal components in such a (suitable) representation produces a ranked ordering of the most relevant (principal component eigenvector) combinations to describe (and differentiate) those shapes. In this representation, particle surfaces are sampled by their projected contours, and the presence of correlated typical nanoscale “features” (*e.g.*, spikes and bumps) dominates the principal component description.

Each structure may then be represented by a single point in a low (often two or three) dimensional representation in the space of major principal component directions, representing an easily understood “shape space”. As an example, several well-known shape types (formerly named as stars, flowers, *etc*.) are easily differentiated as separated clustered “shape space” identities (Figure 1C). We can also determine an “average” shape and capture the largest particle-to-particle ensemble shape fluctuations (deviations) from that mean, thereby quantifying shape polydispersity in different directions in principal component space (Figure 1D), providing a rather complete description of the shape ensemble. This information can now be used to guide the optimization of flow reactor design and parameters until an appropriate level of reproducibility, “purity”, and dispersion of shape distributions has been achieved.

Since biology is sensitive to nanoscale shape, the question of particle shape dispersity is important. Two nearby distributions with different mean shapes may still contain numbers of near identically shaped biologically active particles, making it is necessary to determine how different two (statistically shape independent) ensembles are from each other. In Figure 1E–G we visually represent the issue of shape-distinctiveness between two distributions by comparing the spread in their shape distributions (projected onto the line connecting the two mean shapes) with the “distance” between the mean shapes. It is also possible to numerically quantify the fraction of particles common to both and to stipulate a threshold for independence. These points illustrate the type of shape characterization required for interoperable, reproducible, and reportable nanoscale shape biology. As we show later, they also provide the basis for shape discovery.

### Synthesis of Nanoscale Shapes for Biological Application

Defined shape ensembles typically result from kinetically controlled growth around supercritical spherical-symmetry-broken nuclei (or “seeds”).^6,32−37^ Those seeds possess different crystal growth faces which can be differentially grown by control of the growth kinetics at the different interfaces.^32,38,39^ Consequently, shape-ensemble-growth control features include the density and geometry of the growable (and quenchable) surfaces represented by the seeds,^40−42^ the nature of the reaction (composition and reactants),^24,43^ the rate at which reactants can be deposited at the growing interface, and the nature and amount of the surface-active substance enhancing (“catalyzing”) or inhibiting (“blocking”) the growth kinetics at those surfaces.^44−47^ As slow mixing heterogeneities occur on time scales comparable (or greater) to interface growth kinetics, macroscopic reaction vessels limit our control of shape synthesis.^48,49^ High reproducibility and tunability of shape ensembles require small mixing-volume flow chemistries in which suitable reactor control parameters provide fixed and reproducible constraints between mass transport, interface growth, and quenching kinetics.^50−53^

**Figure 2:**
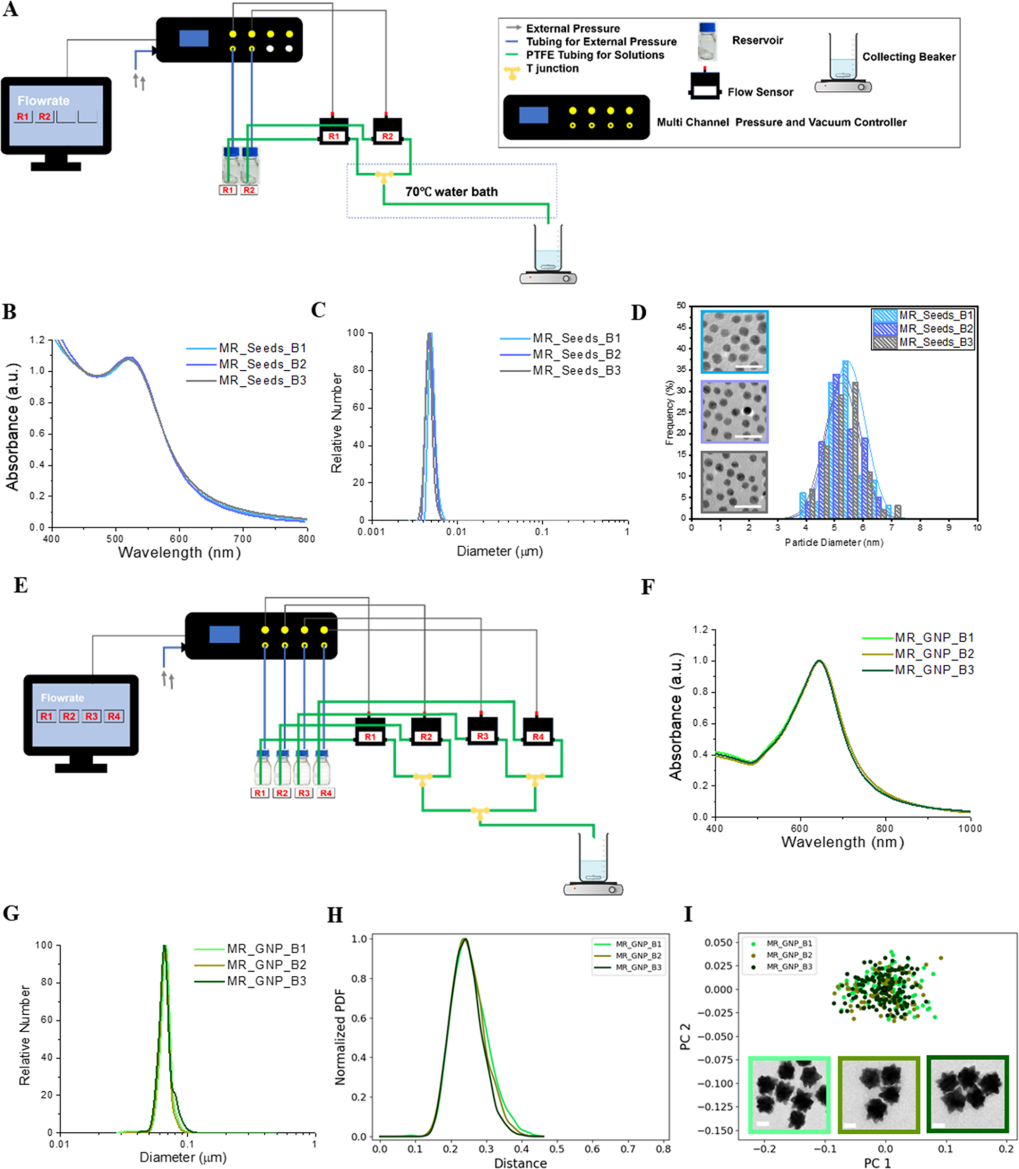
Microfluidic reactor (MR) which can achieve high reproducibility and narrow shape distribution for 5 nm seeds and GNPs. (A) Diagram of the microfluidic reactor synthesis setup for 5 nm seeds. (B) Normalized UV–vis–NIR spectra absorption. (C) DCS analysis showing the high reproducibility of different batches of 5 nm MR_Seeds. (D) Representative TEM micrographs and TEM size distribution. The scale bar is 20 nm. (E) Diagram of the microfluidic reactor synthesis set up for MR_GNPs. (F,G) Normalized UV–vis–NIR spectra absorption and DCS analysis showing the high reproducibility of different batches of MR_GNP. (H) Shape variance expressed as a probability distribution function (PDF) over distance showing the similarity of three batches of MR_GNP. (I) 2D scatter plot of the first two principal components and representative TEM micrographs for each batch MR_GNP. The scale bar is 50 nm.

Here, we employed a fast-mixing microfluidic reactor to tune across a large range of nanoscale shapes while achieving a high shape ensemble reproducibility. Our flow reactor consists of a Luer Lock T-junction, microfluidic sample injection shut-off valves, flow sensors, PTFE tubes (to allow the mixing and further reactions), reaction reservoirs for reagents, and a computer for real-time monitoring and control (Figure 2A, detailed microfluidic synthesis protocol and potential scalability are discussed in supporting method). The reactor leads to highly reproducible symmetry-broken gold seeds (Figure 2B–D). For instance, we observe that it is almost impossible to resolve between three batches of independently produced 5 nm gold seeds using any macroscopic measurement (*e.g.*, UV–vis–NIR absorption spectra and differential centrifugation sedimentation (DCS) shown in Figures 2B and C). Benchtop tank reactor-based synthesis methods do have the advantage of being able to make much more material, but efforts to reproduce this seed type using macroscopic syntheses are challenging and typically lead to numerous failures before suitable batches are made (a typical example is shown in Figure S1).

Starting with high quality and reproducible seeds, we now synthesize a wide range of nanoparticle shapes using various reconfigurable microfluidic setups. As an example, a reactor, including four reservoirs, growth feed solution HAuCl_4_·3H_2_O in reservoir 1, seeds dispersed in trisodium citrate dihydrate (Na_3_Cit) in reservoir 2, and reducing solution hydroquinone in reservoirs 3 and 4 (Figure 2E), was used to synthesize a family of branched gold nanoparticles. The resulting particles drain into the collection vessel containing the initial surface agent of interest (small exchangeable molecules, proteins, other biopolymers or surface-active agents) where the reaction is quenched, and nanoparticles remain dispersed. When proteins are used to quench the system, we observed that, beyond a critical protein concentration, different choices of surface quenching proteins lead to very modest changes in the final shape distribution (Figure S2). The reproducibility of shape dispersions across independent microfluidic syntheses is confirmed by analyses of localized surface plasmon resonance (Figure 2F) and DCS size distribution (Figure 2G). These results are consistent with the well-overlapped shape variance profiles (Figure 2H) and two-dimensional shape scatter plots (Figure 2I). Together, these results illustrate the quite general capacity of such flow reactors to reproduce “typical shapes” accompanied by reproducible and narrow distributions around that average (a comparison of the shape distribution between tank reactor- and flow reactor-based synthesis methods is shown in Figure S3). We note carefully that many flow reactors operate on complex and different principles, some of which are not fully understood as yet.^53−56^ Therefore, it is essential to tune parameters and quantitatively compare the output shape distributions, rather than (only) replicate reactor design.

### Inductive Navigation along Shape Space Trajectories of Biological Interest

Recognizing the unlimited numbers of potential shapes, and complete lack of *a priori* knowledge on which shapes are of biological interest, unguided combinatorial search screening would not be efficient. Therefore, to develop the selection of interesting shape-space regimes we propose an inductive approach. Using a flow reactor, we iteratively make small changes in the reaction parameters, represent the shape ensemble in principal component shape space, and then at appropriate check points, use a cellular readout to decide if we are moving toward (or away from) an interesting area of shape space. Thus, we can build a “shape learning trajectory” by simultaneously varying (single and multiple) combinations of different flow reactor parameters, using shape computation to calibrate the scale of the shape-space increments required to reach the next shape ensemble along the trajectory. There are some noteworthy features associated with searching across shape space, derived from the fact that we are comparing distributions, not simply fixed shapes. For instance, since this is essentially an experimentally based “gradient optimization” search process, when exploring such shape learning trajectories, if we make overlarge changes in the flow reactor parameters in a single step the change in typical shape is discontinuous and it is difficult to discern which aspect (“direction”) of the flow reactor leads to the desirable structural features or specific biological outcomes. That information therefore may not be helpful in choosing which direction to take in the next move. However, there is also a trade-off between the need for these close (small change) points along the learning trajectory that contribute to directional guidance and the fact that nearby distributions have significant “overlaps” corresponding to numerous similar structures being common to the two distributions.

Next, at appropriately chosen steps along this sequence of synthesis-characterization points, we use biological readouts to select useful directions (“slopes”) in the shape-space. Biological readouts from such contiguous distributions (containing many common structures) do not contribute to useful biological information, so computational methods of shape characterization must be used to identify when (along the trajectory) there is sufficient independence to result in significant difference in biology. This inductive shape learning process culminates in a useful “lead location” in shape space. Evidently, all of these steps can (and will be) implemented *via* automated machine learning to optimize the shape-learning process. Here, as a proof of concept we seek to show how the essential elements of the discovery process may be framed. Since we apply the approach manually, on a relatively limited scale (and seek *in vivo* immunological readouts), we use a high-dimensional (“transcriptome”) read-out to choose directions along the learning trajectory.

**Figure 3:**
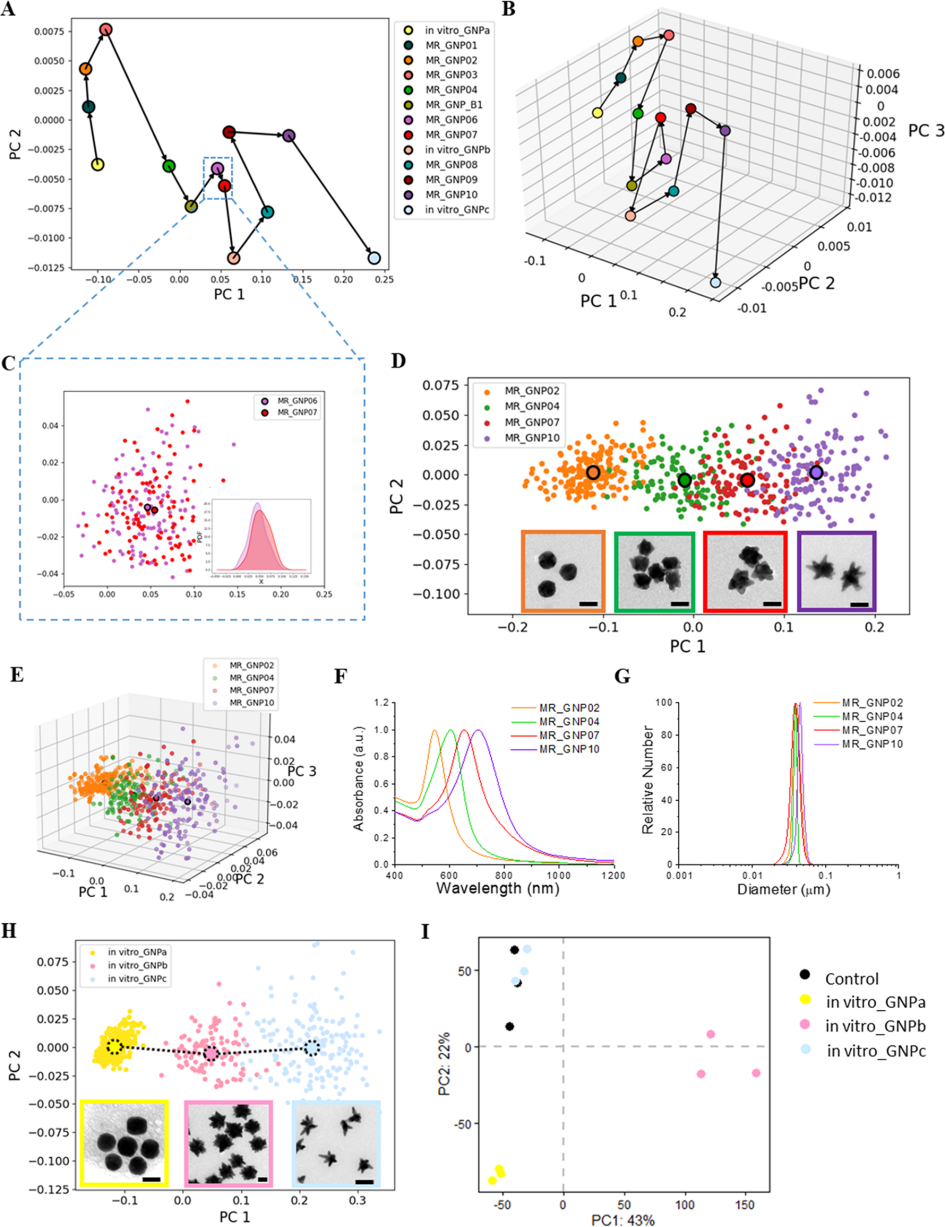
Inductive navigation by microfluidic synthesis along shape space trajectories of biological significance. (A,B) 2D and 3D scatter plots of the first two and three principal components, showing the center of gravity of each shape ensemble to illustrate the “shape learning trajectory”. The arrows indicate the shape-tuning direction. The synthesis methodology for the shape trajectory is reported in the Supporting Information. (C) 2D scatter plot of the first two principal components for MR_GNP06 and MR_GNP07. The inset shows the overlap quantification for these two shapes. (D,E) 2D and 3D scatter plots of the first two principal components for four different shapes. The larger dots with black borders represent the center of gravity of each shape distribution. The insets show TEM micrographs of each shape. The scale bar is 50 nm. (F) Normalized UV–vis–NIR spectra absorption showing the LSPR (localized surface plasmon resonance) of different shapes. (G) DCS analysis showing a similar size distribution of different shapes. (H) 2D scatter plot of the first two principal components for three distinct shape ensembles used in the previously reported transcriptome study.^7^ The larger dots with black dash borders represent the center of gravity of each shape distribution. The insets show representative TEM micrographs for each shape. The scale bar is 50 nm. (I) Principal component analysis illustrating distinctively different transcriptome profiles induced by the three shape ensembles. The percentages shown in the axis labels represent the variance explained by each PC.

Given the fact that we are dealing with a multidimensional, highly nonlinear, and *a priori* unknown relationship between flow reactor parameters and output shape, increments in flow parameters along the shape learning trajectory have to be locally adapted to the regime being explored (Figure 3A,B). For instance, different flow parameter settings (parameter details in Table S1) associated with MR_GNP06 and MR_GNP07 lead to distributions that are barely different statistically (Figure 3C). Many of those along the rest of the trajectory are sufficiently independent to be of interest but in some cases represent such large steps in shape that it is necessary for the shape-learning process to return close to the previous coordinates to recommence more smooth progressions. It is also worth noting that while highly congruent (“nearby”) shape ensembles are useful to understand shape-space tuning in synthesis, meaningful structure–function relationships rely on an independent (investigator-controlled) variable involving the evolution of shape identity along which the (dependent variable) biological read-outs can be measured. For instance, the shape trajectory (Figures 3D,E), in distinction to a shape learning trajectory, is composed of nearly independent ensembles, and biological readouts from these will reflect fundamental changes in shape and would constitute the basis of a useful “structure–function” relationship. Along this trajectory, each particle shape ensemble is labeled by a different color, large dots with black borders represent the “typical” (mean) shape, and illustrations of the overlaps are given in various examples by confidential ellipses (Figure S4). The associated physiochemical properties, including surface plasmon resonance (Figure 3F), effective size distribution of different shapes (Figure 3G), hydrodynamic diameter, and zeta potential (Table S2), are shown.

To direct the trajectory illustrated in Figure 3A we use principal component analysis (PCA) of the whole transcriptome data reported previously^7^ to identify the key changes in biological responses. Those outcomes then help us choose the direction of the learning trajectory to achieve a target biological outcome. Mouse dendritic cells (JAWSII) were treated with the three distinct shapes (marked *in vitro*_GNPa-c, Figure 3H) along the shape-learning trajectory (the details of synthesis and PCA of the transcriptome are described in the Supporting Information.). The transcriptomic changes are captured in the principal component analysis (Figure 3I), illustrating that the transcriptome of *in vitro*_GNPc treated cells returned to the untreated transcriptome where the adjacent shape (*in vitro*_GNPb) gave rise to a distinct transcriptome from the untreated one. It suggests a sharply shape-responsive regime of interest (*e.g.*, region between *in vitro*_GNPb and *in vitro*_GNPc). In summary, despite the fact that the intrinsic nonlinear relationships between shape and flow reactor conditions combined with the complex dependence of biological readouts on shape make it far from obvious how to *a priori* tune shape for biological outcomes, the inductive process outlined here converges relatively quickly along the directions of primary interest.

We next sought to illustrate these concepts in a scientifically interesting, challenging, and practically important shape regime within an *in vivo* setting. This example also illustrates the practical role of computational shape characterization in the “hand-over” of target shape distributions between synthetic approaches (including benchtop tank reactor-based synthesis) that may make the whole workflow, including scale-up, feasible for extended *in vivo* studies.

### Decisive Biological Readouts for Nanoscale Shape

**Figure 4:**
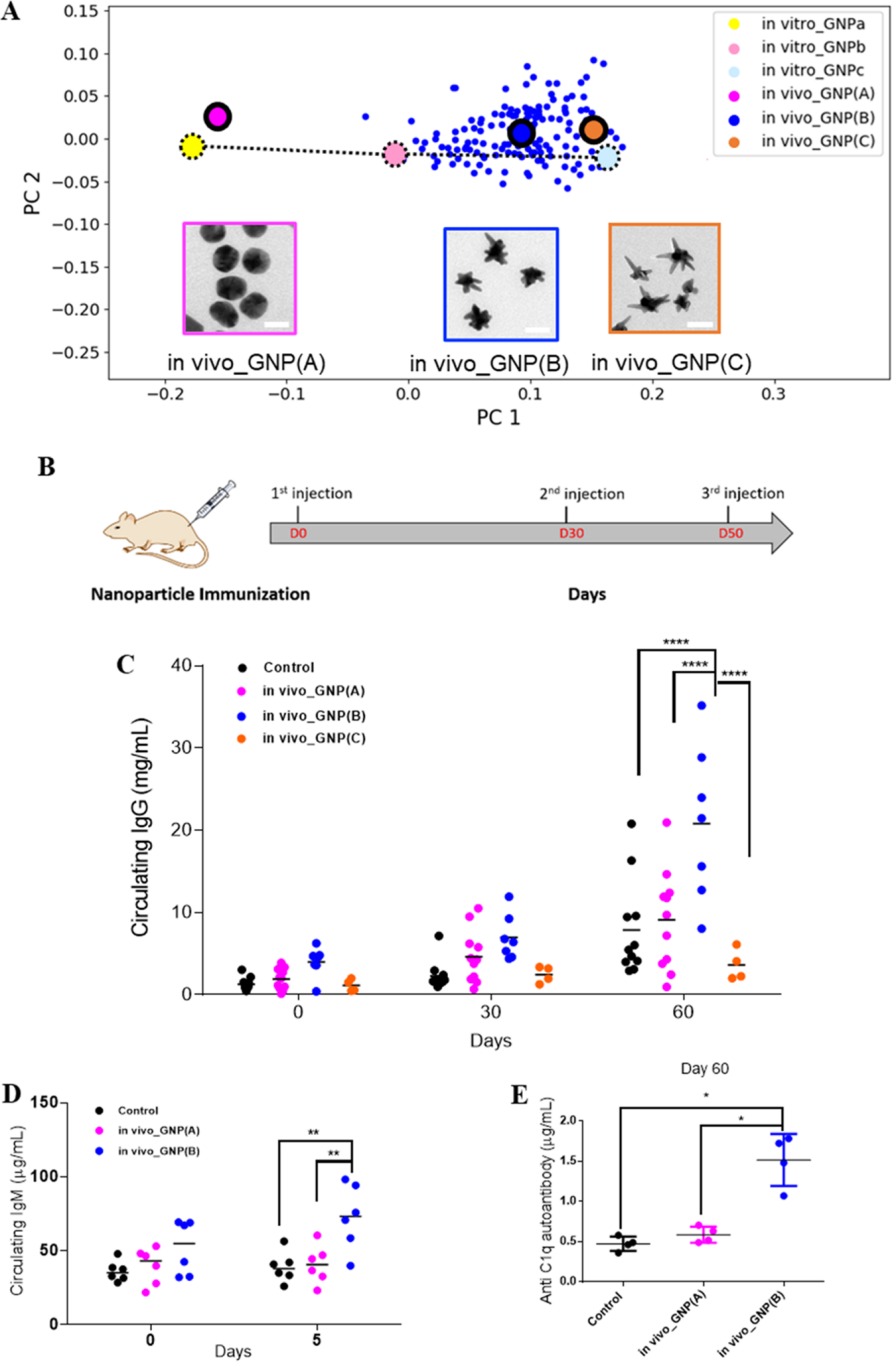
Antibody responses to nanoscale shape ensembles. (A) 2D scatter plot showing the shape distribution of *in vivo*_GNPs shape ensembles in relation to the biological responsive shape regime identified by *in vitro*_GNPs. The larger dots with black borders represent the center of gravity of each shape distribution. Representative TEM micrographs of each shape are shown, and the scale bar corresponds to 50 nm. (B) Subcutaneous immunization schedule in rats. (C) Levels of circulating IgG determined by ELISA. Data are presented as dot plots of individual rats, showing the mean of duplicates. Statistical significance was determined by two-way ANOVA analysis using the Tukey’s test, ****p < 0.0001. (D,E) Circulating IgM and anti-C1q autoantibodies induced by *in vivo*_GNP(A) and *in vivo*_GNP(B) were evaluated by ELISA. Data are presented as dot plots of individual rats, showing the mean of duplicates. Statistical significance was determined by ANOVA analysis using the Tukey’s test, *p < 0.05, **p < 0.01.

Here, we illustrate the larger “shape discovery” potential of the inductive screening approach described by searching for specific adjuvant-like shape-controlled immune responses. We have investigated the “lead” shape ensembles (using large-scale batches, defined *via* their shape geometry) to allow *in vivo* investigation of the shape region of interest. After optimization particle batches (*i.e.*, *in vivo*_GNP(B)) were prepared that occupy the target shape region of interest (*i.e.*, region between in *vitro*_GNPb and in *vitro*_GNPc) (Figure 4A), being of an acceptable structural quality and lipopolysaccharide (LPS) free (the full characterization is shown in Figure S5). The other two shape ensembles (*i.e.*, *in vivo*_GNP(A) and *in vivo*_GNP(C)) with the gravity center of shape distribution shifted away from the target shape region were used as the control. Healthy rats were subcutaneously injected with the same number of gold nanoparticles (Figure 4B). The concentration of serum IgG at Day 0, 30, and 60 (determined by ELISA) was observed to have a general gradual increase of circulating IgG for all the groups over the immunization course, with the steepest rise of the *in vivo*_GNP(B)-treated group (Figure 4C). At Day 60, the concentration of circulating IgG was shown to be approximately 2- to 3-fold higher in *in vivo*_GNP(B)-treated rats than the *in vivo*_GNP(A)- and *in vivo*_GNP(C)-treated groups, respectively. In contrast, the *in vivo*_GNP(C) treated-group exhibited comparable levels of IgG to the control and *in vivo*_GNP(A)-treated groups (Figure 4C). To ensure the accuracy of circulating IgG concentration, we carried out the ELISA measurements by independent operators with each assay performed in replicates (some examples are shown in Figure S6).

Higher IgG levels are prevalent in many autoimmune diseases, such as systemic lupus erythematosus (SLE) and rheumatoid arthritis (RA),^57^ and since the shape ensembles studied here had no exogenous biological antigen, the origin of this rise in IgG levels was explored further for such effects. We assayed serum IgM from Day 0–7 and for several autoantibodies in the *in vivo*_GNP(B)-treated group. The concentration of IgM at Day 5 was found to be about 2-fold higher than Day 0 in the *in vivo*_GNP(C) group, whereas the *in vivo*_GNP(A) treatment or control did not exhibit significant change over the time (Figure 4D). To ensure reproducibility, we repeated the ELISA measurements with independent operators (some examples are shown in Figure S7). One of the autoantibodies against complement C1q (anti-C1q) consistently showed a 3-fold elevation in ELISA for the *in vivo*_GNP(B)-treated group, in contrast to unchanged levels of anti-C1q antibodies observed in the control and *in vivo*_GNP(A)-treated groups (Figure 4E).

Changes in B cell tolerance are often associated with an increase in B cell clonal diversity, resulting in increased self-reactivity,^58^ and B cell clonal diversity is also an important proxy for BCR and antibody repertoires. We therefore harvested the draining lymph node B cells and analyzed the B cell receptor (BCR) repertoire using next-generation sequencing. It was shown that *in vivo*_GNP(B) treatment resulted in a significantly more diverse BCR repertoire than the control and *in vivo*_GNP(A)-treated groups (Figure S8, bioinformatic analysis parameter details in Table S3), consistent with the elevation of autoantibodies. Evidently, these results are quite striking, suggesting a role for nanoparticle shape in the over-riding of usual autoreactive controls. Certainly, under usual circumstances, the frequency and affinity of autoreactive B cells is highly regulated through multiple mechanisms at several sites, including central tolerance in the bone marrow and peripheral tolerance in the spleen, lymph nodes, and other tissues. While B cell tolerance is centrally enforced, leaky self-reactive mature naïve B cells are sometimes found in the periphery,^59^ and their fate is subject to a number of microenvironment contextual signals (such as the specifics of antigen presentation, innate signaling (*e.g.*, TLRs), dendritic cell input (*e.g.*, BAFF), and T cell collaboration (*e.g.*, CD40L)). We therefore hypothesize that exquisitely controlled nanoparticle shape ensembles are able to modulate those contextual signals and lead to a form of self-recognition. While it is not the purpose of this paper to enter into detailed mechanistic investigations of such phenomena, we consider these results constitute a striking example of a definitive readout for nanoscale shape regulation and the potential for inductive discovery processes.

## Conclusion

In this paper, we propose a generally applicable framework that will enable the discovery of important biological and medical outcomes in nanoscale shape biology while supporting the systematic unraveling of the nanoscale shape-dependent biological mechanisms. Our purpose was to highlight and illustrate that these two mutually supportive agendas of “discovery” and systematic mechanistic investigation can be achieved within the same conceptual framework and *via* workflows that are now firmly within the realm of well-defined scientific investigation. Here we chose to illustrate the ideas with an important example of nanoscale shape control that could lead to key practical outcomes.

To assist future researchers in carefully framing directions, here we point out some of the open technical questions. First, we should reiterate carefully that the purpose of the digitization and analysis of shape reported here is not to exhaustively describe the members of the population, though that could be another interesting direction (for instance, in refinement of fluidic syntheses). Rather, our purpose is to find minimal relevant parametrizations that represent useful control parameters to search different types of biological responses, with perhaps some specific target such as vaccine adjuvancy in mind. These two objectives raise quite different questions and require different thinking. Thus, our hypothesis that shape features on the nanoscale can regulate key elements of biology does not preclude the fact that features on other scales or other properties (not monitored in the current type of approach) also affect the outcome, and that has yet to be studied in depth. We should also ask if any particular description (“basic set of descriptors”) we choose is both definitive and sufficient. These are more subtle questions than they may appear at first sight. For instance, in a particular class of material shapes and syntheses a given parametrization may appear to be sufficient to adaptively improve the design in the narrower sense. However, given the loss of detail (say in going from two-to-three-dimensional representations) in the description we cannot preclude the outcome that other materials with similar projected information may have similar biology. Also, one cannot *a priori* exclude the fact that, for instance, other synthetic setups could appear to give the similar projected information but not have the same effects, simply because the descriptor chosen by the researcher is insufficient to fully describe the biology. While that might seem unlikely, there remains the possibility (especially for larger search spaces) that the really key biological control features are (within a certain synthetic framework and material class) “slaved” (correlated) to the characteristics we describe, and we have not yet isolated the fundamental parameters. Again, our approach may be sufficient in a specific study but may require enlargement for the field to develop. Of course, such questions can be resolved by evolution of a more complete set of shape characterizations (even the use of three-dimensional information) and parametrizations. These are quite deep questions that can only be resolved by accumulation of knowledge and many more well-chosen examples.

We also stress that much has yet to be achieved to understand the detailed mechanisms in shape control biology. The issues are subtle, and it would be premature to make definitive statements on that topic. For instance, we have known for some time that it is the composition and collective organization of the surface biomolecular (corona) layer, rather than only individual surface molecules, that are recognized by nanoscale biological mechanisms specifically evolved for that purpose.^1−3^ And it is that collective recognition that determines many early and later downstream biological outcomes. There are certainly common features between that corona paradigm (largely based on multiple and simultaneous molecular motif engagements between the nanoscale surface and cognate cell receptors and other interacting membrane proteins) and repetitive features on various particle geometries, such as those reported here. Indeed, potentially spatially correlated nanoshape features could be recognized and transduced at the cell membrane as patterns of differentiated stress relaxation but could also (at least to some degree) be coupled to collective surface molecular recognition. That issue of decoupling the surface and shape effects is subtle and will take time to clarify.

It will be necessary to acquire detailed and elaborated evidence to reliably assign mechanisms of shape regulation *in vivo*, including those shape-induced self-immune responses discussed here. Still, practically speaking, our observations of *in vivo* shape regulation are potentially highly significant, suggesting the possibility of controlling the breaking of balances of immunity and tolerance. We note carefully that such effects may point toward an avenue to develop adjuvants and immunotherapies possessing local regulatory functions without affecting systemic immune tolerance. The implications for significantly improved safety and efficacy in vaccine applications are clear. In a scientifically related issue, the results reported here also raise questions related to the environmental impact of processes producing rich varieties of nanoscale shape fragments. To confirm such a link to human health and autoimmune disease it would be necessary to carefully and fully investigate a variety of material types, conditions, and species in the ecosystem. However, given the long-standing suspicion that autoimmune diseases are linked to environmental dusts, the issues involved appear significant and should be investigated.^60−62^ In that context, we note carefully that the difficulty in locating these effects (indeed requiring inductive searching) may suggest such autoimmune-shape biology effects are not ubiquitous and may be confined to highly specific shapes. If it is true, then the good news is that their limited nature could make conceivable their isolation and elimination, with significant implications for human health. Shape searches using the full machinery of inductive shape learning (or otherwise very insightful hypotheses) to isolate and identify these effects may therefore constitute a practical, feasible, and scientifically well-founded frontier in hazard identification for environmental health investigations.

However, these considerations all represent specific tasks that should be considered by science. The central purpose of this paper was to create conceptual and practical order out what at first sight looks like a nanoscale shape cacophony. That is to create a systematic shape-discovery framework that will lead to interesting discoveries. We believe that automation and implementation of machine learning of the framework of inductive shape discovery presented in this paper will play a central role in the search for valuable (or elimination of harmful) shape space entities. Simultaneously, it will allow the shape-biology-medicine research enterprise to be moved onto well-established scientific principles, enabling widely shared reproducible and validated results from scientific research. That will frame a secure basis on which to pursue future research in the field.

## Methods

### Chemicals

The following chemicals were purchased from Sigma-Aldrich and were of highest available purity and used as received: hydrogen tetrachloroaurate trihydrate (HAuCl_4_·3H_2_O, ≥99.9%), trisodium citrate dihydrate (C_6_H_9_Na_3_O_9_, meets USP testing specifications), potassium carbonate (K_2_CO_3_, ≥99%), tannic acid (C_76_H_52_O_46_, ACS Reagent grade), hydroquinone (HQ, C_6_H_6_O_2_, ≥99%), silver nitrate (AgNO_3_, ≥99.9%), glycerol (C_3_H_8_O_3_, ≥99%), sucrose (C_12_H_22_O_11_, ≥99.5%), dodecane (CH_3_(CH_2_)_10_CH_3_, ≥99%), clean water (CHROMASOLV Plus, for HPLC), bis(*p*-sulfonatophenyl)phenylphosphine dihydrate dipotassium salt (BSPP, C_18_H_17_K_2_O_8_PS_2_, 97%), bovine serum albumin (BSA, lyophilized powder, ≥98%), human serum albumin (HAS, lyophilized powder, ≥98%), ovalbumin (OVA, lyophilized powder, ≥98%). Sodium hydroxide (NaOH, ACS Reagent grade) was purchased from Fluka. Poly(vinyl chloride) (PVC) calibration standard for differential centrifugal sedimentation (DCS) measurements (263 nm) was purchased from Analytik Ltd.

### Microfluidics

Oil-free air compressor (8 bar) and Luer Lock T-junctions (microfluidic manifold three-port small kit) were purchased from Darwin microfluidics. The other microfluidic equipment and adjuncts were purchased from Elveflow including: microfluidic flow controller (OB1MK3+, channel pressure range 0–8 bar), microfluidic flow sensors (MFS, flow rate range 0–5 mL/min), PTFE tubing (1/16 in. OD × 1/32 in. ID, 50 m), microfluidic reservoir for 100 mL bottles (bottle cap with two 1/4 in. 28-threaded ports), and microfluidic fittings (1/4 in. 28 thread).

### Computational Shape Analysis

To avoid aggregation due to drying effects and to obtain well-dispersed imaging of nanoparticles (NPs), sample preparation for TEM imaging used a modified method based on the previously reported protocol.^7^ Grids (Agar Scientific) were pretreated with a glow discharger, and 1 μL of 1 × 10^10^–1 × 10^11^ NPs/mL sample solution was deposited on the grid. Imaging was performed using a FEI Tecnai G2 20 Twin TEM at 200 kV, with magnifications no less than 19000×. TEM images containing well-spread NPs were used to extract their contours following the protocol previously reported by our group.^7^ The obtained contours were then used to analyze the shapes of the different batches of NPs.

### Nanoparticle Immunization

All rat work was performed in accordance with institutional guidance, the NIH Guide for the Care and Use of Laboratory Animals (2011 edition), and EU directives and guidelines (EEC Council Directive 2010/63/UE). Adult CD (Charles River) male rats (approximately 300 g in body weight) were housed paired in individually ventilated cages (Tecniplast S.p.A., Varese, Italy) and maintained under specific pathogen-free conditions in the Institute’s animal care facilities. They received food and water *ad libitum* and were regularly checked by a certified veterinarian responsible for animal welfare supervision and experimental protocol revision. The investigators were not blinded to allocation during experiments and outcome assessment. To exclude contamination, all the procedure concerning animals were performed in a Class 2 laminar flow hood following strict precautions. The reagents used for NP preparation were opened inside the laminar flow fume hood. Immediately before the treatment, NPs were dissolved in water to reach a concentration of 1.5 × 10^12^ NP/mL in a final volume of 500 μL and injected subcutaneously into the loose skin over the interscapular area. NPs and water were administered at day 1 (first boost), at day 30 (second boost), and at day 50 (third boost). Rats were randomly assigned to the following treatment groups: control (*n* = 11 for IgG ELISA; *n* = 6 for IgM ELISA), *in vivo*_GNP(A) (*n* = 11 for IgG ELISA; *n* = 6 for IgM ELISA; *n* = 4 for anti-C1q autoantibody ELISA), *in vivo*_GNP(B) (*n* = 7 for IgG ELISA; *n* = 6 for IgM ELISA; *n* = 4 for anti-C1q autoantibody ELISA), *in vivo*_GNP(C) (*n* = 4 for IgG ELISA).

### Blood Collection and ELISA

Under general anesthesia (continuous flow of 5% isoflurane/oxygen mixture for induction and 2–3% for maintenance), blood was taken from the lateral tail vein at each indicated time point. Day 0 is defined as the day of injection. Blood on Day 0 was taken before the injection. Blood was collected into EDTA-tubes and centrifuged for 15 min at 1500*g* at 4 °C. The supernatant (plasma) was aliquoted and immediately frozen. Animals were sacrificed on Day 60. Blood was collected through a terminal cardiac puncture, and then animals were euthanized by CO_2_ inhalation.

The levels of circulating IgG and IgM in rat plasma were determined by Ready-SET-Go! total rat IgG and IgM ELISA (cat. no. 88-50490 and 88-50540, eBioscience) with predilution of 1:250 000 and 1:5000 in the provided sample diluent for IgG and IgM, respectively. The concentration of autoantibody anti-C1q IgG was determined by rat anticomplement 1q antibody ELISA (cat. no. MBS722996, MyBioSource, USA) with predilution of 1:10 in the provided sample diluent.

## Supporting Information Available

Detailed synthesis and characterization methods for GNPs used in the paper; (Figure S1) characterization of 5 nm seeds synthesized by benchtop method; (Figure S2) characterization of MR_GNPs with different protein coatings; (Figure S3) shape distribution comparison between flow reactor and benchtop tank reactor-based synthesis; (Figure S4) shape distribution of MR_GNPs illustrated by confidential ellipses; (Figure S5) characterization of GNPs used for *in vivo* study; (Figure S6) replicates of IgG expression for *in vivo*_GNP(A) and *in vivo*_GNP(C); (Figure S7) replicates of IgM expression for *in vivo*_GNP(A) and *in vivo*_GNP(B); (Figure S8) comparison of clonal diversity between repertoires by Hill diversity curves using Change-O; (Table S1) synthesis recipe for GNPs1–10; (Table S2) hydrodynamic diameter and zeta potential of MR_GNPs; (Table S3) detailed bioinformatics analysis parameters (PDF)

## Notes

Animal ethics: The animal work was reviewed by the IRCCS-IRFMN Animal Care and Use Committee (IACUC) and approved by the Italian “Istituto Superiore di Sanità” (code: 42/2016-PR).

## Notes

Code and data availability: The data sets generated and analyzed during the current study are available in the Figshare repository, https://doi.org/10.6084/m9.figshare.17429657. The deposit is entitled: Supplementary data set for “A Nanoscale Shape-Discovery Framework Supporting Systematic Investigations of Shape-Dependent Biological Effects and Immunomodulation”—a submission to ACS Nano.
