# Supplementary material for: A Nanoscale Shape-Discovery Framework Supporting Systematic Investigations of Shape-Dependent Biological Effects and Immunomodulation
Source: ACS Nano. 2021 Dec 27;16(1):1547–59. doi: 10.1021/acsnano.1c10074 (PMC8793145; doi:10.1021/acsnano.1c10074)
Supplement: Supplementary file 1 — nn1c10074_si_001.pdf [file nn1c10074_si_001.pdf]

Supporting Information:

A Nanoscale Shape-Discovery Framework Supporting Systematic Investigations of Shape-Dependent Biological Effects and Immunomodulation

Wei Zhang,[†,‡,§] Hender Lopez,[‡,&,§] LucaBoselli,[‡,§] Paolo Bigini,[//,§] André Perez-Potti[‡,§] Zengchun Xie,[‡,§] Valentina Castagnola,[‡] Qi Cai,[‡] Camila P. Silveira,[‡] Joao M. de Araujo,[‡,⊥] Laura Talamini,[//] Nicolò Panini,[//] Giuseppe Ristagno,# Martina B. Violatto,// Stéphanie Devineau,[‡] Marco P. Monopoli,[‡] Mario Salmona,[//] Valeria A. Giannone,[‡] Sandra Lara,[‡] Kenneth A. Dawson,[,†,‡] and Yan Yan,[*,‡,g]*

[†]Guangdong Provincial Education Department Key Laboratory of Nano-Immunoregulation Tumor Microenvironment, The Second Affiliated Hospital, Guangzhou Medical University, Guangzhou 510260, Guangdong P.R. China

[‡]Centre for BioNano Interactions, School of Chemistry, University College Dublin, Belfield, Dublin 4, Ireland

[&]School of Physics and Optometric & Clinical Sciences, Technological University Dublin, Grangegorman D07XT95, Ireland

[ǁ]Istituto di Ricerche Farmacologiche Mario Negri IRCCS, Via Mario Negri 2, 20156 Milan, Italy

[⊥]Departamento de Física Teórica e Experimental, Universidade Federal do Rio Grande do Norte, 59078970 Natal, RN, Brazil

[#]Department of Pathophysiology and Transplantation, University of Milan, 20122 Milan, Italy

[g]School of Biomolecular and Biomedical Science, UCD Conway Institute of Biomolecular and Biomedical Research, University College Dublin, Belfield, Dublin 4, Ireland

*Email: Kenneth.a.dawson@cbni.ucd.ie; yan.yan@cbni.ucd.ie

§. These authors contributed equally: Wei Zhang, Hender Lopez, Luca Boselli, Paolo Bigini, André Perez-Potti and Zengchun Xie

Supporting methods

Synthesis of TR_Seeds and MR_Seeds

TR_Seeds were synthesized *via* a previously reported method[1]. Briefly, TR_Seeds were prepared by adding a reducing solution (10 mL) containing trisodium citrate (0.068 mmol, 6.80 mM), tannic acid (0.003 mmol, 0.29 mM) and potassium carbonate (0.013 mmol, 1.25 mM) to 40 mL of gold precursor ($HAuCl_4 \cdot 3H_2O$ 0.013 mmol, 0.32 mM) at 60 °C under vigorous stirring. The mixture was then heated to reflux for 2-3 min and consequently cooled to room temperature. The pH was adjusted to 8.5 using NaOH (500 mM). TR_Seeds were filtered with a 0.2 μm filter before use.

For the synthesis of MR_Seeds, 50 mL of $HAuCl_4 \cdot 3H_2O$ (2.8×10^{-5} mol, 0.56 mM) was placed in reservoir 1 (R1, inlet 1), 50 mL of reducing agent containing trisodium citrate (8.2×10^{-5} mol, 0.4 mM), tannic acid (3.5×10^{-6} mol, 0.07 mM), and potassium carbonate (1.5×10^{-5} mol, 0.3 mM) was placed in reservoir 2 (R2, inlet 2). The reagents were mixed in the T-junction at 70°C and the outlet tubing was also immersed in a 70°C water bath during the synthesis. The flow rate was 2700 μL/min. The tubing length between the reservoir and flow sensor was 30 cm, the tubing length between the flow sensor and T-junctions was 10 cm and the total length of outlet tubing was 10 m. 50 mL of the resulting particles were collected in a 250 mL beaker and the particles were stirred to cool to room temperature. 500 mM NaOH solution was used to adjust the dispersion pH to 8.5 before characterization. MR_Seeds were filtered with a 0.2 μm filter before use.

Synthesis of TR_GNP and MR_GNPs

TR_GNP was synthesized by the same method as described for *in vivo*_GNP(A), details are discussed below in the synthesis of *in vivo*_GNPs section. By using the high reproducibility and homogenous size distributed MR_Seeds, we succeeded in synthesising high reproducibility branched MR_GNPs using a similar microfluidic set up. Briefly, four reservoirs containing 50 mL of reagents were used. Four flow sensors were placed between reservoirs and T-junctions to monitor the flow rate. All of the flow rate was set as 2700 μL/min. The reaction took place in PTFE tubing and T-junctions. The tubing length

between the reservoir and flow sensor was 30 cm, the tubing length between the flow sensor and T-junctions was 10 cm and the total length of outlet tubing was 15 m.

MR_GNP_B1-B3 (characterization shown in Figure 2F-I) synthesis in the microfluidic system was initiated through the mixing of three solutions: growth solution (reservoir 1), seeds solution (reservoir 2) and reducing solution (reservoir 3 and 4). 5×10^{-5} mol, 276 mM $HAuCl_4\cdot3H_2O$ was added in reservoir 1, 1.5×10^{-5} mol, 1% w/v sodium dihydrate citrate and 300 μL MR_Seeds was added in reservoir 2, and 2×10^{-4} mol, 200 mM hydroquinone was added to reservoirs 3 and 4, respectively. The resulting particles were collected in a beaker with BSA (1 mg/mL, 10 mL) and stirred at 37 °C for 30 mins.

For the synthesis of MR_GNP01-10, all the microfluidic set up parameters remained the same, the tuning of shapes was achieved by changing the reagents quantities as shown in Table S1.

UV-Vis-NIR spectroscopy

All UV-Vis-NIR spectra were recorded on an Agilent Cary 6000i UV-Vis-NIR spectrophotometer in the range 400-1200 nm. The measurements were carried out using a quartz cell with a path length of 1 cm.

Differential centrifugal sedimentation (DCS)

DCS experiments were run using a CPS instrument (DC24000). An 8-24% sucrose gradient either water-based or phosphate-buffered saline (PBS)-based (particles with protein coating) was used. Calibration was performed using polyvinyl chloride (PVC) particles (0.263 μm, Analytik Ltd.), following successful PVC standard calibration, 0.1 mL of the sample was injected for analysis.

Transmission electron microscopy (TEM)

Samples for TEM imaging were prepared by evaporating ca. 10 μL of the nanoparticle suspension onto formvar-coated copper grids (Agar Scientific). Imaging was performed by using FEI Tecnai G2 20 Twin TEM and data analysis using ImageJ. Images for computational shape analysis were taken by a specific method mentioned in the main text computational analysis part.

Quantification of the overlap between two NP shape distributions

To quantify the overlap between two NP shapes, we first perform a principal component analysis (PCA) of the shape descriptors (Fourier coefficients) as explained in detail in our previous paper[2]. Once the PCA is calculated, the pair of NP groups for which the overlap is being studied is represented in the 3D space corresponding to the first three PCs. The line that joins the centre-of-gravity of the two groups will be considered the axis in which to calculate the shapes distribution and which we label as X. The "clouds" corresponding to the PCs of the NPs of the two groups are then projected onto X. Finally, the probability distribution function of the projected points on X are calculated and the overlap of the shape distributions can be represented.

Calculation of the principal components for the shape learning trajectory

The PCA for the shape space trajectories was performed as explained in our previous paper[2] but only the data from the initial and final shapes are used in the calculation of the eigenvectors during the PCA. In this way we obtained a projection matrix, which projects the Fourier coefficients into the PC space defined by the initial and final shapes. Then, the calculated projection matrix is used to project all the intermediate shapes into this PC space.

Transcriptome

The synthesis of *in vitro*_GNPa-c was reported previously[2]. In brief, *in vitro*_GNPa and *in vitro*_GNPb were synthesized using a microfluidic method, and *in vitro*_GNPc was synthesized by a benchtop method. After synthesis, the three types of GNPs were dispersed in 1 mg/mL OVA to coat the particle surface with OVA.

Transcriptome experiment details were reported in our previous work[2]. JAWS II cells were treated with 3×10^{10} *in vitro*_GNPs for 24 h. After the treatment, the cells were washed with PBS, and subsequently the total RNA was extracted by InviTrap® Spin Universal RNA Mini Kit. Each treatment was performed in replicates and repeated independently three times. The transcriptome was analyzed by using Whole-Mouse Genome One-Color Microarray (Agilent). After correcting the background and

filtering low intensity signals, data were expressed in the mean of replicates in a log2 scale. PCA was performed by R studio (v.4.0.3) and the additional package ggplot2.

Synthesis of *in vivo*_GNPs

*In vivo*_GNPs were prepared in water *via* the seed-mediated method reported previously[1]. For *in vivo*_GNP(A), gold seeds were prepared by adding 4.5 mL of trisodium citrate (0.15 mmol, 34 mM) to 150 mL of a boiling gold precursor solution ($HAuCl_4 \cdot 3H_2O$, 0.038 mmol, 0.25 mM). The mixture was stirred under reflux for 30 min and overnight at room temperature. 12 mL of the seeds and 1.53 mL of trisodium citrate (0.052 mmol, 34 mM) were added to 300 mL of aqueous solution of $HAuCl_4 \cdot 3H_2O$ (0.089 mmol, 0.30 mM) under reflux and then stirred for 30 min. After a further addition of trisodium citrate (11.8 mL, 0.400 mmol, 34 mM) the mixture was stirred under reflux for a further 1 h, then cooled to room temperature and filtered through 0.2 μm filters. 200 mg of BSPP (0.38 mmol) were dissolved in 5 mL of water and added to 150 mL of the prepared NP suspension and stirred overnight. The sample was then washed several times using centrifugal filters (10000 MWCO, 600 rcf) using water with low BSPP concentration (4 μM) and concentrated.

For *in vivo*_GNP(B) and *in vivo*_GNP(C), 0.5 mL of the TR_Seeds described above was added to 100 mL 4 °C ultrapure water containing 0.5 mL of glycerol, 0.1 mL of $HAuCl_4 \cdot 3H_2O$ (0.022 mmol), 0.5 mL $AgNO_3$ solution (0.0005 mmol, 1 mM) and ascorbic acid (0.5 mL, 0.05 mmol, 100 mM) were subsequently added under vigorous stirring. After 30 s 1.0 mL of BSPP solution (0.311 mmol, 311 mM) was added and the mixture was gently stirred at room temperature overnight. The dispersion was finally filtered through 0.2 μm filters and washed.

In order to avoid lipopolysaccharide (LPS) contamination, all the synthetic processes for *in vitro* and *in vivo*_GNPs were performed using the standard operational procedures associated with cell culture. All the processes were carried out in a laminar flow cabinet (Class II, type B2) located in a laboratory with filtered air and limited access. All the solvents and the reagents were strictly opened inside the laminar flow cabinet after purchase and tested for LPS contaminant. All the plasticware used (*e.g.* falcon tubes, Eppendorf tubes, pipette tips, syringes, *etc.*) were endotoxin-free certified. All the glassware used in the reaction were previously soaked in fresh aqua regia for at least 30 mins, and then carefully rinsed with

endotoxin-free water. The LPS level of GNPs were detected by Limulus Amebocyte Lysate (LAL) assay before using.

Lymphatic B cell isolation

Armpit lymph nodes were extracted from treated animals and transferred to Dulbecco's Modified Eagle Medium (DMEM) Glutamax (GIBCO, 10566-016) supplemented with 10% heat inactivated fetal bovine serum (HI-FBS) on ice and processed immediately after extraction. The same organs from the same treatment group were pooled and mechanically homogenized by disrupting them through a 70 µm cell strainer (Thermo Fisher, 22363548) using a plunger of a sterile syringe until no more tissue was left. Cells were collected in 50 mL Falcon tubes with 50 mL DMEM 10% HI-FBS and split into 2 tubes. Cells were spun down for 10 min at 1300 rpm for further erythrocyte lysis. Erythrocyte lysis was performed using the erythrocyte lysing kit (WL2000) following the manufacturer's instructions for each of the resulting tubes from each organ and treatment group. Non-erythrocytic cells were resuspended in 5 mL DMEM with 10% HI-FBS carefully and transferred to a new tube. Cells were counted in order to further perform the B cell isolation.

B cell isolation was performed by means of the MagCellect B cell isolation kit (MAGR303). Two aliquots of 2×10^8 cells from the spleen homogenates and whole lymph node homogenates were used. Cells were spun down at 1300 rpm for 5 minutes and resuspended in 1 mL of ice-cold 1X MagCellect Buffer. 200 µL of MagCellect Rat B Cell Biotinylated Antibody cocktail was added for negative selection of B cells and incubated at 4°C for 15 minutes. 250 µL MagCellect Streptavidin Ferrofluid were added to the cell suspension and incubated at 4°C for 15 minutes. After that, 1.55 mL 1X MagCellect Buffer was added. Samples were applied to a magnetic stand for 6 minutes at room temperature and the supernatant containing B cells was extracted. This step was repeated to ensure removal of all tagged cells.

Total RNA extraction and cDNA library preparation

B cell total RNA isolation was performed using the Invitrap® Spin cell RNA mini kit (10061100300) following the manufacturer's instructions. In the case of the B cells isolated from the spleens,

purification was done from 2 aliquots of 1×10^7 B cells, while for the lymph nodes, only 1 aliquot containing all the B cells was performed. RNA quality and concentration were checked immediately after the purification by Nanodrop. 1 µg and 150 ng of purified RNA from spleen and lymph nodes, respectively, of each group were used for retrotranscription RNA, at concentrations of 50 and 7.5 ng/µL respectively. High-capacity cDNA reverse transcription kit (4368814) was used for RT-PCR, and the 2X master mix was prepared as follows: 4.0 µL 10X RT buffer, 1.6 µL 25X dNTP Mix, 4.0 µL 10X RT Random primers, 2.0 µL Reverse Transcriptase, 8.4 µL Nuclease-free water. PCR was performed as follows: 25°C for 10 minutes; 37°C for 60 minutes; 37 °C for 60 minutes; 85°C for 5 minutes; 4°C. 4 µL of cDNA at 150 and 20 ng/ µL for spleen and lymph nodes were used from each sample to amplify the corresponding Ig specific variable regions. Amplification of Ig specific rat variable amplicons were amplified following a previously reported primer mix targeting the framework regions 1 and 4. A total of 50 PCR reactions were performed for each of the samples (*i.e.* different experimental group and organ). Twenty-four reactions for heavy chain amplification (each reaction contained 1 heavy chain specific forward primer + combination of 4 heavy chain backward primers) and 26 reactions for light chain amplification (each reaction contained 1 light chain specific forward primer + combination of 5 light chain specific backward primers). Q5® High-Fidelity DNA Polymerase kit (M0491L) was used following the suggested PCR master mix by the manufacturer.

PCR was performed as follows: 90°C for 5 minutes; 35 cycles of: 95°C for 30 seconds, 60°C for 30 seconds, 72°C for 30 seconds; 72°C for 10 minutes. Final PCR products were mixed and cleaned by a AMPure XP PCR purification Kit (A63880) using a ratio of beads of 0.8X of the total volume of the mixture and elution was done in 40 µL of 10 mM tris-acetate pH 8.0. Illumina Nextera indexes were included for Next Generation Sequencing using the Nextera XT Illumina Index Kit (FC-131-1096). For each group, 3 aliquots of 12.5 µL (containing 100 ng/µL of cleaned variable region amplicons) a short cycle PCR for attachment of indexes was performed following manufacturer's instructions. PCR was performed as follows: 72°C for 3 minutes; 92°C for 30 seconds; 12 cycles of: 95°C for 10 minutes, 55°C for 30 seconds, 72°C for 30 seconds. The clean-up procedure was performed as before, and amplicons were eluted in 40 µL of 10 mM tris-acetate pH 8.0. Quality and concentration were checked by Bioanalyzer prior to sequencing and 30 µL at 5 nM were used for sequencing.

V(D)J Repertoire analysis

IMGT/High-VQUEST alignment files for the different groups were analyzed by means of the tools provided in the Immcantation (V2.5.0) portal (https://immcantation.readthedocs.io/en/version-2.5.0/), consisting of the packages Change-O, Alakazam, SHazaM and TIgGER for BCR repertoire analysis.

References

1. Talamini, L.; Violatto, M. B.; Cai, Q.; Monopoli, M. P.; Kantner, K.; Krpetić, Ž.; Perez-Potti, A.; Cookman, J.; Garry, D.; Silveira, C. P.; Boselli, L.; Pelaz, B.; Serchi, T.; Cambier, S.; Gutleb, A. C.; Feliu, N.; Yan, Y.; Salmona, M.; Parak, W. J.; Dawson, K. A.; *et al*. Influence of Size and Shape on the Anatomical Distribution of Endotoxin-Free Gold Nanoparticles. *ACS Nano* 11, 5519–5529 (2017).

2. Boselli, L.; Lopez, H.; Zhang, W.; Cai, Q.; Giannone, V. A.; Li, J.; Moura, A.; de Araujo, J. M.; Cookman, J.; Castagnola, V.; Yan, Y.; Dawson, K. A., Classification and Biological Identity of Complex Nano Shapes. *Commun. Mater.* **2020,** *1, 1-12.*

Supporting Figures

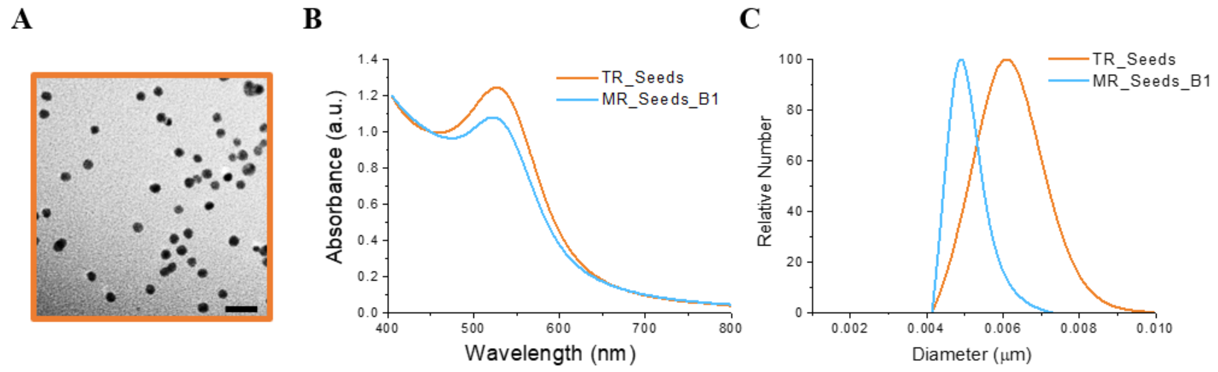

Figure S1. Characterization of 5 nm seeds synthesized by benchtop tank reactor (TR_Seeds).

(A) TEM micrographs for TR_Seeds, scale bar is 20 nm. (B) Normalized UV-Vis-NIR absorption for TR_Seeds and MR_Seeds. (C) DCS analysis showing a narrower size distribution of MR_Seeds compared with TR_Seeds.

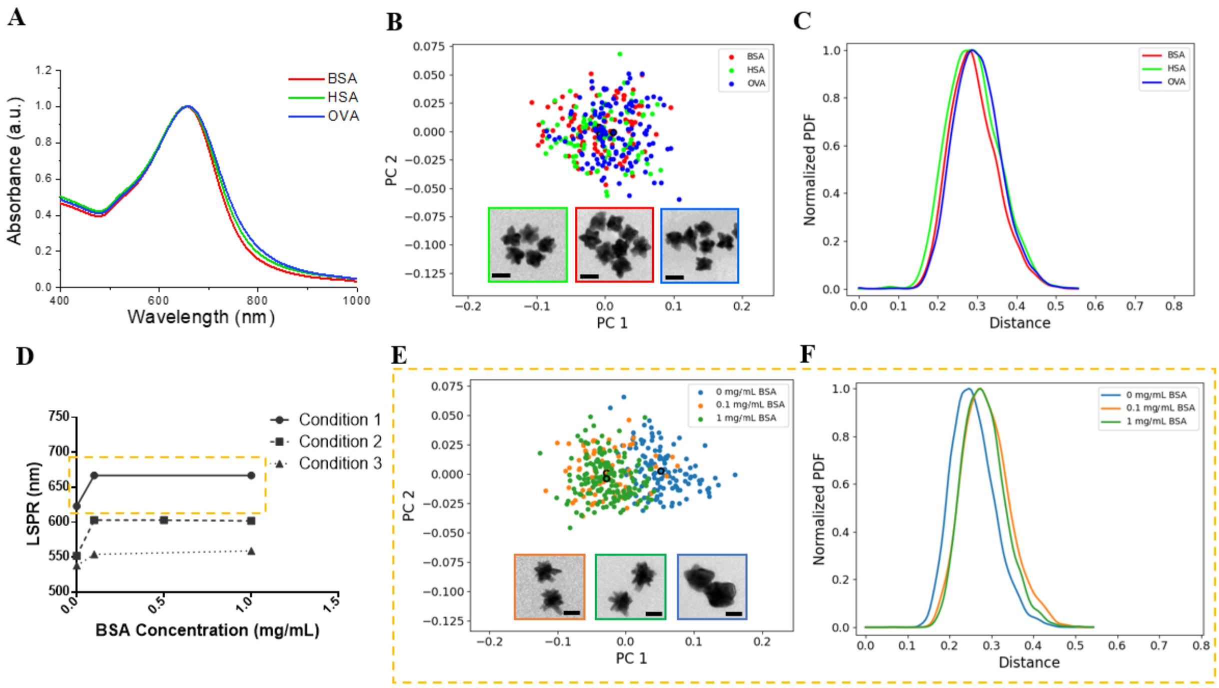

Figure S2. Characterization of MR_GNPs with different protein coatings.

(A) Normalized UV-Vis-NIR absorption showing similar localization surface plasmon resonance (LSPR) of different protein associated shape synthesis, bovine serum albumin (BSA), human serum albumin (HSA) and ovalbumin (OVA) concentrations are 1 mg/mL. (B-C) 2D scatter plot, representative TEM micrographs and shape variance showing similar shape and shape distribution for different protein coating GNPs. (D) LSPR corresponding to BSA concentration plot showing the protein amount effect on shape, condition 1 is 400 µL seeds, 1 × 10^{-4} mol hydroquinone and 3 × 10^{-7} mol $AgNO_3$; condition 2 is 400 µL seeds and 4 × 10^{-4} mol hydroquinone; condition 3 is 200 µL seeds and 3 × 10^{-5} mol hydroquinone. $HAuCl_4{\cdot}3H_2O$ (5 × 10^{-5} mol) and sodium dihydrate citrate (1.5 × 10^{-5} mol) are kept the same for condition 1-3. (E-F) 2D scatter plot, representative TEM micrographs and shape variance for condition 1. Scale bar is 50 nm.

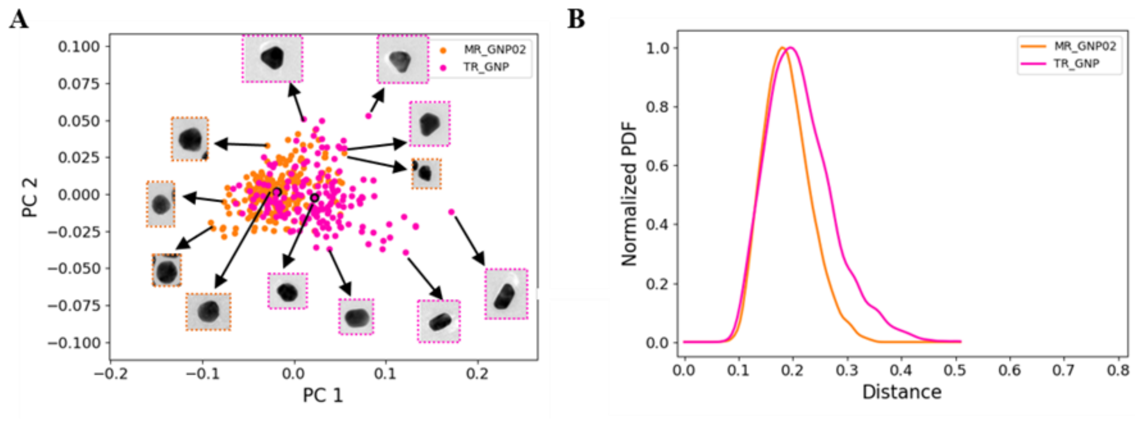

Figure S3. Shape distribution comparison between flow reactor- (MR) and benchtop tank reactor (TR)-based synthesis.

(A-B) 2D scatter plot and shape variance showing more homogenous and narrower shape distribution of MR_GNP02.

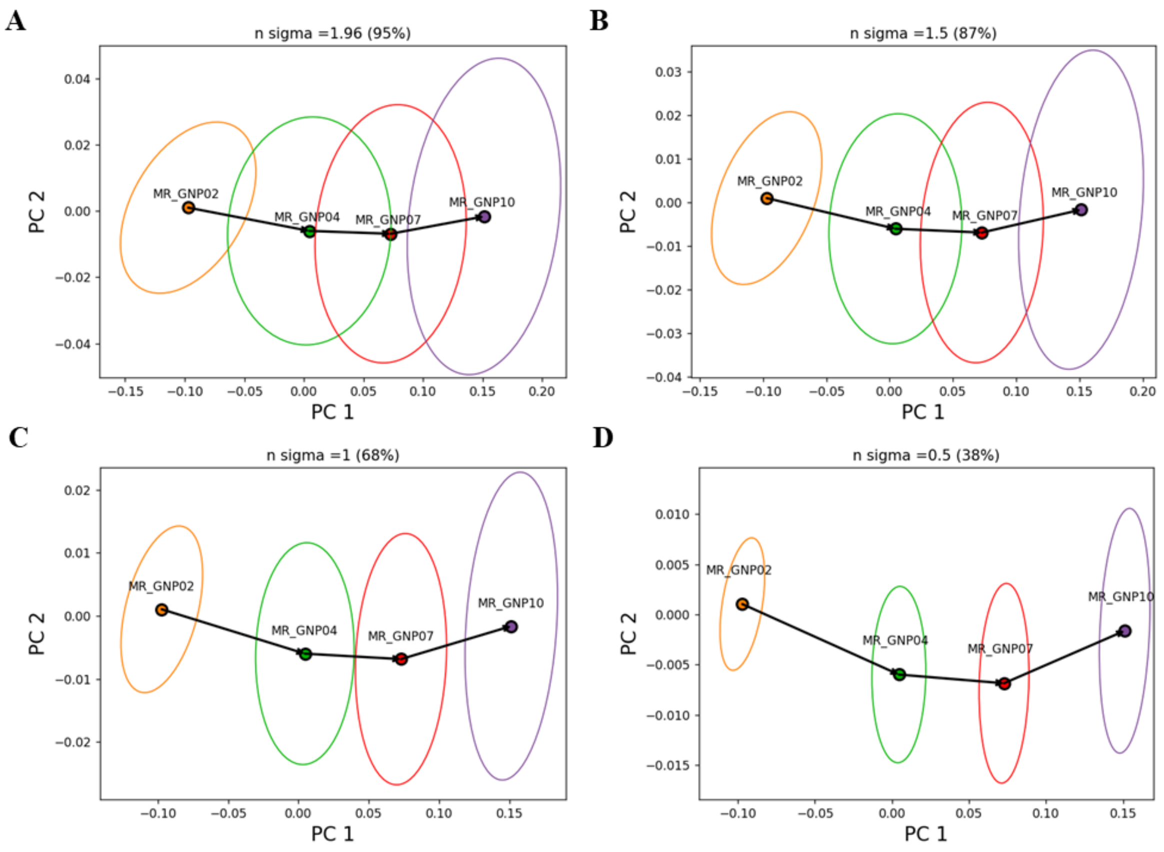

Figure S4. Shape distributions of MR_GNPs illustrated by confidential ellipses.

In each panel an ellipse represents the likelihood (interval of confidence) that a certain percentage of the points are contained in that ellipse. The axis of each ellipse is obtained as follows. We assume that the distribution of points for all groups are normally distributed. Based on this assumption we then calculate the variance and orientation of the each of the gaussians for each group on the 2D PC space. Finally, an ellipse with certain number of standard deviations (sigma) of the distributions previously calculated is drawn. For example: n sigma = 1.96 (95%) means the ellipse contains 95% of the data outlines with an internal of confidence.

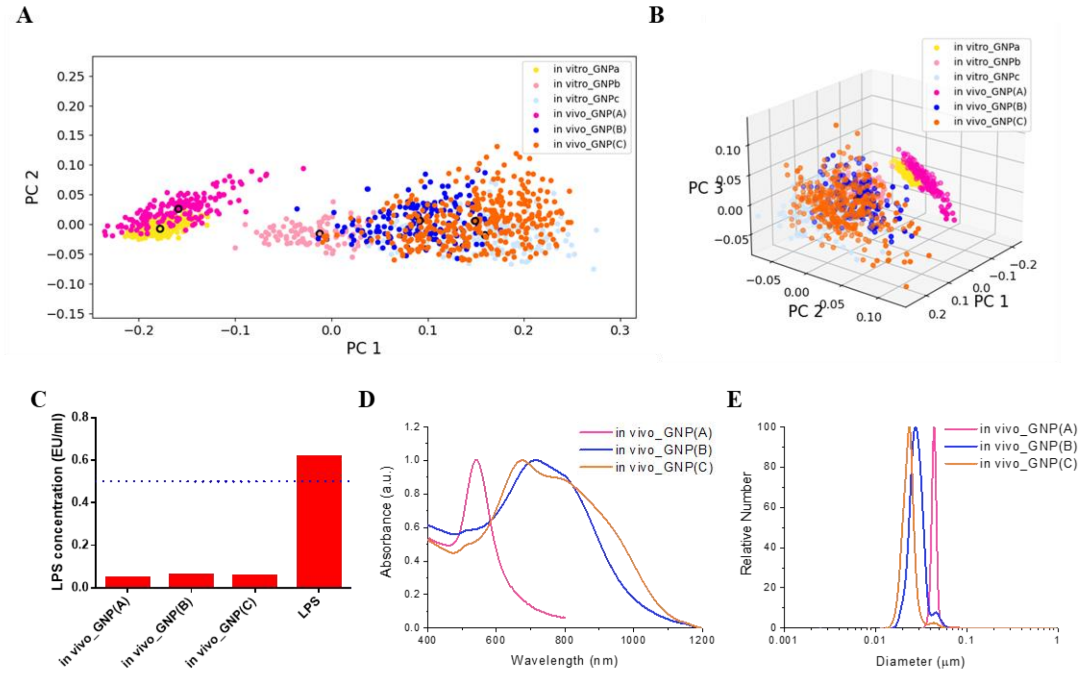

Figure S5. Characterization of GNPs used for *in vivo* study.

(A-B) 2D and 3D scatter plot for *in vitro*_GNPs and *in vivo*_GNPs, (C) LPS level of *in vivo*_GNPs, (D) Normalized UV-Vis-NIR absorption of *in vivo*_GNPs, (E) DCS size distribution analyzed by relative number representing the size distribution of *in vivo*_GNPs.

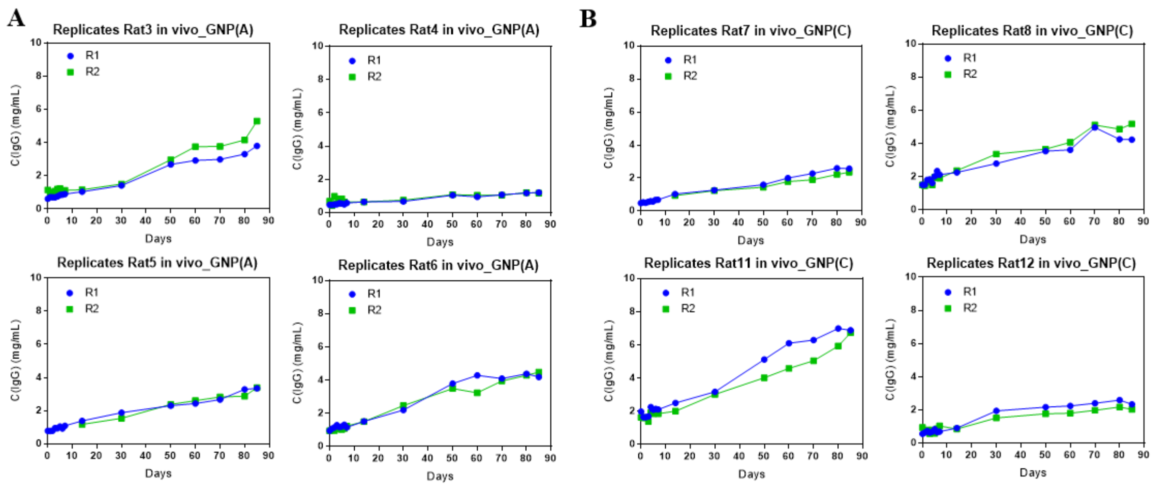

Figure S6. Replicates of IgG expression for *in vivo*_GNP(A) and *in vivo*_GNP(C).

Data are the mean of duplicates. R1 and R2 present two different operators.

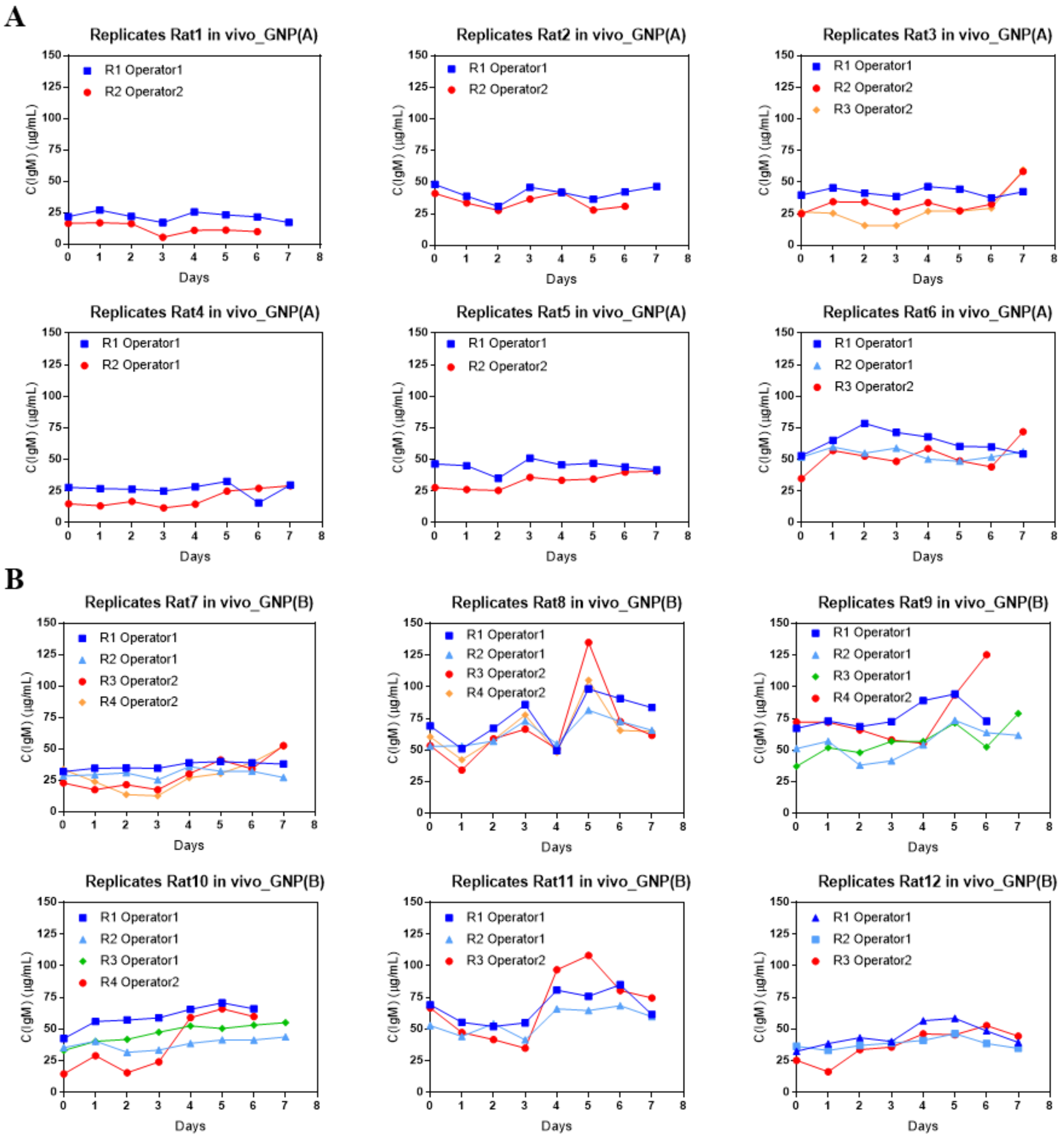

Figure S7. Replicates of IgM expression performed by different operators for *in vivo*_GNP(A) and *in vivo*_GNP(B).

Data are the mean of duplicates. R1 and R2 present two different operators.

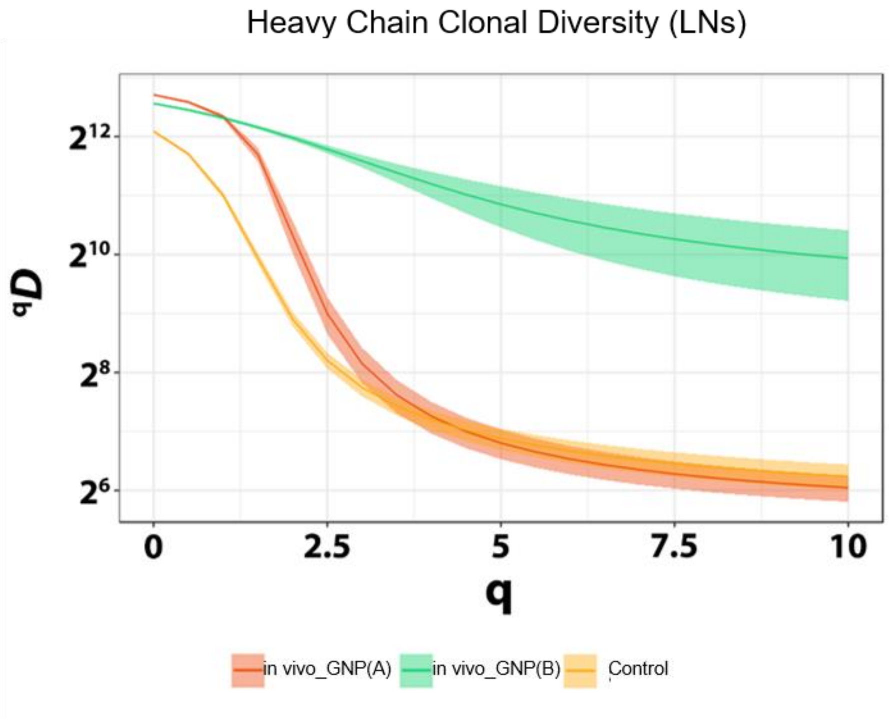

Figure S8. Comparison of clonal diversity between repertoires by Hill diversity curves using Change-O.

Diversity indexes (^qD) were estimated by uniform sampling of the lymphatic B cell repertoires across different diversity orders (q).

Reservoirs / GNPs	R1			R2		R3	R4
	$HAuCl_4·3H_2O$	$AgNO_3$	Glycerol	MR_ Seeds	Na_3Cit	HQ	HQ
MR_GNP01	$5×10^{-5}$ mol	0	0	400 μL	$1.5×10^{-5}$ mol	$3×10^{-5}$ mol	$3×10^{-5}$ mol
MR_GNP02	$5×10^{-5}$ mol	0	0	300 μL	$1.5×10^{-5}$ mol	$3×10^{-5}$ mol	$3×10^{-5}$ mol
MR_GNP03	$5×10^{-5}$ mol	0	0	200 μL	$1.5×10^{-5}$ mol	$3×10^{-5}$ mol	$3×10^{-5}$ mol
MR_GNP04	$5×10^{-5}$ mol	0	0	300 μL	$1.5×10^{-5}$ mol	$4×10^{-4}$ mol	$4×10^{-4}$ mol
MR_GNP06	$5×10^{-5}$ mol	0	0	300 μL	$1.5×10^{-5}$ mol	$1×10^{-4}$ mol	$1×10^{-4}$ mol
MR_GNP07	$5×10^{-5}$ mol	$5×10^{-8}$ mol	0	300 μL	$1.5×10^{-5}$ mol	$1×10^{-4}$ mol	$1×10^{-4}$ mol
MR_GNP08	$5×10^{-5}$ mol	$1×10^{-7}$ mol	0	300 μL	$1.5×10^{-5}$ mol	$1×10^{-4}$ mol	$1×10^{-4}$ mol
MR_GNP09	$5×10^{-5}$ mol	$3×10^{-7}$ mol	0	300 μL	$1.5×10^{-5}$ mol	$1×10^{-4}$ mol	$1×10^{-4}$ mol
MR_GNP10	$5×10^{-5}$ mol	$1×10^{-7}$ mol	500 μL	300 μL	$1.5×10^{-5}$ mol	$1×10^{-4}$ mol	$1×10^{-4}$ mol

Table S1. Synthesis recipe for MR_GNP01-10. Reaction volume for each reservoir is 50 mL.

	Hydrodynamic diameter (nm)	Polydispersity index (PDI)	Zeta potential in H_2O (mV)
MR_GNP02	51 ± 1	0.15 ± 0.01	-28 ± 1
MR_GNP04	50 ± 1	0.12 ± 0.01	-24 ± 1
MR_GNP07	49 ± 1	0.20 ± 0.01	-24 ± 1
MR_GNP10	57 ± 1	0.08 ± 0.01	-26 ± 2

Table S2. Hydrodynamic diameter and zeta potential of MR_GNPs.

Action	Tool/Program	Parameters
Read processing	Trimmomatic V0.32	ILLUMINACLIP:2:30:10<br>LEADING:3<br>TRAILING:3<br>MINLEN:36<br>NexteraPE-PE.fa (adapters)
Paired-End read alignment	PEAR V0.9.8	-p 0.05 -v 15 –q 30 –n 300 –m 500
V(D)J germline alignment	IMGT/High-VQUEST	Species:Rattus norvegicus<br>locus:IG<br>Single individual:NO
IMGT output formatting for Immcantation tools: Parsing	Change-O "MakeDb"	--partial true<br>--scores true<br>--regions true<br>--junction true
IMGT output formatting for Immcantation tools: Removing non-functional	Change-O "ParseDb"	select<br>-f FUNCTIONAL<br>-u T
IMGT output formatting for Immcantation tools: Separating heavy and light chains	Change-O "ParseDb"	select
IMGT output formatting for Immcantation tools: Adding group name and organ	Change-O "ParseDb"	add columns with corresponding names
Polymorphism identification	TIgGER "findNovelAlleles"	germLine_min = 100<br>min_seqs = 50<br>auto_mutrange = TRUE<br>mut_range = 1:10<br>y_intercept = 0.125<br>alpha = 0.05
Inferring Genotype	TIgGER "inferGenotype"	default
Correcting allele calls	TIgGER "reassignAlleles"	method = hamming<br>keep_gene = gene
Calculating distance to nearest neighbor	SHazaM "distToNearest"	model = ham<br>normalize = none<br>symmetry = avg<br>first = FALSE

Finding distance threshold for clonal assignment	SHazaM "findThreshold"	method = density
Clonal clustering	Change-O "defineClones"	--mode allele --act set --model ham --dist (calculated before) --sym avg
Reconstruct germline alignments	Change-O "CreateGermLines"	--vf V_CALL_GENOTYPED
V(D)J family and gene usage	Alakazam "countGenes"	groups = GROUP/ORGAN mode = family/gene clone = CLONE
Clonal diversity	Alakazam "rarefyDiversity"	clone = CLONE max_n = 9000 min_q = 0 max_q = 10 step_q = 0.5 ci = 0.95 nboot = 2000 uniform = TRUE

Table S3. Detailed step-by-step bioinformatic analysis with the corresponding parameters for each of the procedures.
